# First New World Primate Papillomavirus Identification in the Atlantic Forest, Brazil: *Alouatta guariba papillomavirus 1*

**DOI:** 10.1128/genomeA.00725-16

**Published:** 2016-08-18

**Authors:** Rodrigo Vellasco Duarte Silvestre, Alex Junior Souza de Souza, Edivaldo Costa Sousa Júnior, Allan Kaio Silva, Wyller Alencar de Mello, Marcio Roberto T. Nunes, João Lídio S. G. V. Júnior, Jedson Ferreira Cardoso, Janaina Mota de Vasconcelos, Layanna Freitas de Oliveira, Sandro Patroca da Silva, Adriana Marques J. da Silva, Brigida Gomes Fries, Maria Eugênia L. Summa, Lilian Rose M. de Sá

**Affiliations:** a Section of Virology, Evandro Chagas Institute, Ministry of Health, Ananindeua, Brazil; b Section of Hepathology, Evandro Chagas Institute, Molecular Biology Laboratory, Ministry of Health, Belém, Brazil; c Center for Technological Innovation, Evandro Chagas Institute, Ministry of Health, Ananindeua, Brazil; d Technical Division of Veterinary Medicine and Wildlife Management, Environment and Green Areas Secretary, São Paulo, Brazil; e Department of Pathology, Veterinary Medicine and Animal Science Faculty, São Paulo University, São Paulo, Brazil

## Abstract

We report here the complete genome sequence of the first papillomavirus detected in a New World primate, howler monkey, *Alouatta guariba clamitans papillomavirus 1* (AgPV1), from the Atlantic Forest in São Paulo State, Brazil.

## GENOME ANNOUNCEMENT

The *Papillomaviridae* family is composed of 49 genera and 316 virus species distributed in human and other animal hosts (1, 2). With an icosahedral capsid and a conserved circular double-stranded DNA, papillomaviruses have genomes ranging from 6,953 bp in *Chelonia mydas papillomavirus* type 1 (CmPV1) to 8,607 bp in *Canis familiaris oral papillomavirus* type 1 (CPV1). These viruses contain eight open reading frames (ORF), with the exception of some species that lack the E5 ORF (1). Primate papillomavirus has been described so far in Old World primates (3–7), but until then, there was one report of papillomavirus identification and lesion described in a howler monkey without complete genome description (8) and one incomplete genome description in squirrel monkey (Z. Chen, C. E. Wood, and R. D. Burk, unpublished data). For this reason, this is the first description of papillomavirus detection in New World primates with complete genome annotation.

We describe here the complete sequencing of the new papillomavirus species that naturally infected a howler monkey, a New World native primate. Howler monkeys (*Alouatta guariba clamitans*) are distributed from the southeastern Brazilian Atlantic Forest to northeastern Argentina (9) and the species was identified in our study according to morphological features. 

Genomic DNA was obtained from a frozen sample of oral mucosa that was previously suggestive to this infection (8) using organic solvent extraction, and precipitation was made with ethanol. The DNA mass used for sequencing was 1 µg in the Ion Torrent platform (Life Technologies). The sequencing procedures generated 281,859 reads that were compared to a public data bank of annotated sequences containing 316 papillomavirus complete genomes using the BLAST program (10). Of the total reads analyzed, 1,049 reads presented similarity to reads in these genomes and were separated, assembled, and ordered by a *de novo* strategy.

The scaffold generated was about 7.04 kb and was used as reference mapping in the final genome assembly. This cycle of assembly was repeated until the genome was completely assembled. The annotation was performed using the 316 papillomavirus genomes as references, and the functional annotation was done manually.

Our final result was the assembly of a genome that differs from the closest species, *Saimiri sciureus* (accession no. JF304765), by 30%. The genome is 7,722 bp in length and is arranged in a double strand, with 7 fully identified genes and a G+C content of 59.2%. The new genome identified had a coverage of about 340×, forming a circular unitig containing the complete genome. These observations correspond with a typical papillomavirus genome and, to our knowledge, this is the first description of a papillomavirus detected in oral mucosa of a New World primate and can be used as a reference for further studies of papillomavirus evolution.

### Nucleotide sequence accession number.

This whole-genome shotgun project has been deposited in GenBank under the accession no. KP861980. The version described in this paper is the first version.
